# Scientific Writing in the Age of Artificial Intelligence

**DOI:** 10.31662/jmaj.2026-0083

**Published:** 2026-06-12

**Authors:** Soichiro Saeki

**Affiliations:** 1 Emergency Medicine and Critical Care, National Center for Global Health and Medicine, Japan Institute for Health Security, Tokyo, Japan; 2 Division of Public Health, Department of Social Medicine, Graduate School of Medicine, The University of Osaka, Osaka, Japan

**Keywords:** artificial intelligence, linguistic diversity, academic publishing, postgraduate education and training, scholarly communication

I read with great interest the recent letter by Matsubara ^(1)^ on maintaining the human voice in scientific writing in the era of artificial intelligence (AI). I fully agree with his concern that overwhelming reliance on generative AI may gradually erode individuality, diversity, and personal tone in medical manuscripts. Writing is not merely a technical process but a reflection of scientific judgment, ethical responsibility, and professional identity.

Beyond individual style, the AI era also raises broader structural questions regarding linguistic diversity and knowledge dissemination. In Japan, some major academic societies have recently expressed concern over the decreasing number of submissions to long-established Japanese-language journals ^(2), (3)^. These discussions underscore the growing tension between global English-language publications and the sustainability of native-language scholarship. Supporting both Japanese- and English-language publishing should be recognized as a strategic investment in Japan’s academic ecosystem, rather than a zero-sum choice.

For authors, writing in their first language facilitates deeper cognitive engagement, clearer logical construction, and more authentic expression of individual voice. Native-language writing, therefore, functions as an effective pedagogical strategy, even in the AI era, by strengthening critical thinking and scientific reasoning. Although AI tools may assist with later translation and editing, they cannot replace this foundational intellectual process ^(4)^.

Moreover, recent advances in machine translation and AI-based literature retrieval have increased the international visibility of non-English publications. When supported by appropriate archiving systems, metadata, and bilingual titles and abstracts, articles written in Japanese can now be discovered, interpreted, and cited beyond national and linguistic boundaries. Native-language scholarship should therefore not be regarded as secondary but as an integral component of global knowledge production.

In this context, the central challenge is to balance human creativity, linguistic diversity, and technological efficiency. Clear editorial policies, guidance on responsible AI use, and training in critical manuscript revision are essential.

On the basis of my experience in academic writing, I share Matsubara’s concern for future generations of authors. Preserving human voice and native-language scholarship is not incompatible with global communication. Rather, it is a prerequisite for sustainable and trustworthy scientific publishing. By integrating thoughtful regulation with educational and structural support ^(5)^, we can ensure that AI enhances, rather than diminishes, the intellectual and cultural richness of medical literature ^(4)^.

## Article Information

### Acknowledgments

Soichiro Saeki thanks his colleagues for helpful discussions on this topic. The author acknowledges the use of Grammarly (Grammarly Inc., San Fransico, USA) for primary language editing. The views expressed in this manuscript are Soichiro Saeki’s own and do not necessarily represent the author’s institutions.

### Author Contributions

Soichiro Saeki is solely responsible for the manuscript content. Artificial intelligence technology was used for language editing, and the content was reviewed by the author. The author’s institution played no role in the conceptualization of this manuscript.

### Conflicts of Interest

None
